# Regulation of glycosylation in radiotherapy: exploring the multiple effects of DNA damage, immune response, stromal microenvironment and metabolism

**DOI:** 10.3389/fonc.2026.1758628

**Published:** 2026-03-19

**Authors:** Wenqing Cui, Mengqian Jiang, Ran Zhang, Jinming Yu, Dawei Chen

**Affiliations:** 1 School of Clinical Medicine, Shandong Second Medical University, Weifang, China; 2 Department of Radiation Oncology, Shandong Provincial Key Laboratory of Precision Oncology, Shandong Cancer Hospital and Institute, Shandong First Medical University, Shandong Academy of Medical Sciences, Jinan, Shandong, China

**Keywords:** DNA damage, glycosylation, immune response, metabolism, radiotherapy, tumor microenvironment

## Abstract

Radiotherapy remains a central component of cancer care, but its clinical benefit is frequently compromised by intrinsic or acquired radioresistance. Growing evidence indicates that glycosylation, one of the most prevalent post-translational modifications, is not merely a bystander but an active determinant of how tumors respond to irradiation. In this review, we organize the literature by separating glycosylation into mechanistically distinct layers—O-GlcNAcylation, N-glycosylation, mucin-type O-glycosylation, and terminal sialylation—and summarize how each layer shapes radiotherapy outcomes through effects on the DNA damage response (DDR), antitumor immunity, stromal remodeling, and metabolic adaptation. Within DDR, dynamic O-GlcNAc cycling governed by OGT and OGA can promote repair signaling and post-irradiation survival. By contrast, changes in N-glycan processing more often affect DDR indirectly, for example by tuning proteostasis and receptor-dependent signaling, and in certain settings through PD-L1 trafficking and functions. In the tumor immune microenvironment, glycosylation influences both checkpoint stability and glycan–lectin interactions (such as sialoglycan–Siglec pathways) that can dampen immunity after radiotherapy. Irradiation can also remodel glycosylation in endothelial cells and the extracellular matrix, with consequences for immune-cell recruitment and fibrotic responses. Finally, radiation-induced metabolic stress may shift nucleotide-sugar availability (including HBP-derived UDP-GlcNAc), linking metabolic state to glycosylation programs and radiosensitivity. We conclude by outlining therapeutic opportunities as well as practical hurdles—such as specificity, toxicity, and delivery—that must be addressed before glycosylation-targeted radiosensitization can be translated to the clinic.

## 1 Introduction

Cancer remains one of the leading causes of death worldwide. According to the International Agency for Research on Cancer, 13 million people are expected to die from cancer in 2030 (1). In recent years, various strategies have been investigated to improve cancer treatment, including surgery, radiotherapy, chemotherapy, immunotherapy, targeted therapy, hormonal therapy, and so on (2). Among them, radiotherapy, as one of the most important ways to control or kill tumors, is widely used in more than 50% of cancer patients (3). Its core mechanism is to directly damage the DNA structure of tumor cells through ionizing radiation, leading to irreversible damage such as double-strand breaks, triggering irreversible cell cycle block or programmed death (4). In addition, radiotherapy can also indirectly affect radiotherapy efficacy by altering the immune microenvironment or tumor angiogenesis (5, 6). However, the DNA repair capacity of tumor cells themselves (e.g., repair enzyme system) may impair radiotherapy efficacy, and increased infiltration of immunosuppressive cells after radiotherapy suppresses anti-tumor immune responses (7). Meanwhile, biophysical factors such as extracellular matrix hardness, interstitial fluid pressure, and so on can alter tumor cell mechanical signals and promote radiotherapy resistance (8). In conclusion, radiotherapy resistance results from the dynamic intertwining of multiple mechanisms, and an in-depth investigation of the molecular mechanisms of radiotherapy resistance and the development of sensitization strategies is essential for cancer treatment.

Glycosylation is one of the most common post-translational modifications of proteins, which can regulate various biological functions by affecting protein folding, transport, and localization (9). There are 14 different types of protein glycosylation, including N-glycosylation, 11 types of O-glycosylation, C-mannosylation, and glycosylphosphatidylinositol (GPI)-anchored protein production, of which N-glycosylation and O-glycosylation are the two most common ones (10). And aberrant glycosylation is considered to be an essential factor in tumorigenesis and development, which can act in various stages of tumorigenesis and development, such as proliferation, apoptosis, invasion, metastasis, and immune evasion (11).

Because glycosylation covers multiple processes that differ in mechanism, subcellular location, and kinetics, we discuss O-GlcNAcylation, N-linked glycosylation, mucin-type O-linked glycosylation, and terminal sialylation as distinct regulatory layers (12). Accordingly, throughout the review we state the specific modification being considered and highlight key context factors—such as radiation dose, fractionation, the timing of sample collection after irradiation, and tumor lineage—that can strongly influence how results should be interpreted (**Table 1**).

**Table 1:** Key features of major glycosylation modalities discussed in this review.

Modality	Localization/machinery	Key RT-relevant mechanisms and endpoints	Representative examples in this review
O-GlcNAcylation	Nucleus/cytosol (also mitochondria); OGT and OGA	Rapid cycling after IR; tunes chromatin signaling (gammaH2AX spread), pathway choice (HR/TLS vs NHEJ), and checkpoint recovery	H2AX; RAD18; YTHDC1; NONO/Ku; p27/cyclin D1
N-glycosylation	ER-Golgi secretory pathway; OST complex (e.g., RPN1/2, STT3A/B, DDOST) and processing enzymes	Often indirect: reshapes RTK/integrin/checkpoint signaling into ATM/ATR/DNA-PK; controls trafficking/stability; links to ER-stress/redox adaptation	PD-L1; ST6GAL1/ST3GAL4 programs; CDON example
Mucin-type O-glycosylation	ER-Golgi; GALNTs and core extension enzymes	Remodels glycocalyx and receptor clustering; affects cell adhesion and immune recognition in RT settings	GALNT2-linked radioresistance and related pathways
Terminal sialylation/fucosylation	Golgi and cell surface; ST3GAL/ST6GAL and FUT enzymes	Modulates glycan-lectin interactions (Siglec engagement), immune suppression, endothelial adhesion, and fibrosis programs after RT	Siglec-10/Siglec-15 axes; endothelial glycome remodeling

In recent years, it has been found that glycosylation plays a vital role in tumor cell response to radiotherapy, and it plays a key role in radiotherapy resistance by regulating DNA damage repair, signaling pathways, tumor microenvironment, and other aspects. Therefore, targeting specific glycosylation modifications to intervene in the regulation of DNA damage in tumor cells has become an important research direction for sensitization to radiotherapy (13). It has also been preliminarily demonstrated that glycosylation inhibitors such as OMSI-1 and OMSI-2, class OGT inhibitors, can improve their sensitivity to radiotherapy (14). Therefore, in this paper, we will systematically review the multiple mechanisms of glycosylation in radiotherapy, including its functions in DNA damage repair, immune response, tumor stromal remodeling, and metabolism, with a focus on the feasibility and research progress of targeting glycosylation as a strategy for sensitization to radiotherapy.

## 2 The role of glycosylation in radiotherapy-induced DNA damage

Radiotherapy can kill tumor cells by high-energy photon radiation (X-rays or γ-rays), causing several forms of DNA damage, including base damage, DNA single-strand breaks(SSB), and double-strand breaks(DSB) (15). Double-strand breaks is considered the most dangerous form of DNA damage, capable of leading to cell death, gene mutation, and chromosomal instability (16). However, DNA damage caused by radiotherapy can effectively kill tumor cells. To survive, tumor cells can respond to radiotherapy-induced DNA damage by slowing down or blocking cell cycle progression and activating DNA damage repair pathways such as non-homologous end joining and homologous recombination (17). However, DNA repair, such as DNA damage response, leads to radioresistance and limits the therapeutic outcome in cancer patients. Therefore, understanding the mechanism of radioresistance may help develop more effective cancer treatments (18, 19).

DDR pathway activation is a key causative factor for radiotherapy resistance. In recent years, it has been found that post-translational modifications of proteins, especially O-GlcNAc glycosylation, can be an emerging hub that affects radiotherapy sensitivity by dynamically regulating DDR core protein function (20). Therefore, exploring the role of glycosylation in DDR and radioresistance, especially the effect of different types of glycosylation on radiotherapy sensitivity, will help to optimize radiation therapy strategies for cancer. Among the many glycosylation modifications, N-glycosylation and O-glycosylation are particularly important. In the following section, this study will examine the mechanisms of action in DDR and radiotherapy response.

### 2.1 Glycosylation in the regulation of DNA damage response

Rather than a single linear pathway, the DNA damage response (DDR) operates through several repair modules—homologous recombination (HR), non-homologous end joining (NHEJ), translesion synthesis (TLS), and checkpoint control—each with its own post-irradiation timing, cell-cycle constraints, and core effector proteins. In the current literature, the most direct evidence for glycosylation acting on DDR comes from rapid, reversible O-GlcNAcylation of nuclear factors at damage sites, driven by O-GlcNAc transferase (OGT) and removed by O-GlcNAcase (OGA) (21–24). Consistent with this, ionizing radiation has been reported to increase global O-GlcNAc levels and to concentrate O-GlcNAc signals at DNA damage foci (22).

It is also important to separate glycosylation processes that operate on different cellular “layers.” O-GlcNAcylation can act directly within the nucleus to tune repair complex assembly and break-associated signaling. By contrast, N-glycosylation in the ER–Golgi secretory pathway usually influences DDR more indirectly—through effects on receptor-dependent survival signaling, protein trafficking, and proteostasis/ER-stress adaptation—which can feed into ATM/ATR/DNA-PK checkpoint regulation (25–28).

#### 2.1.1 HR and TLS: γH2AX-centered O-GlcNAc/phosphorylation interplay, and direct O-GlcNAc modification of RAD18 and YTHDC1

Phosphorylation of histone H2AX (γH2AX) is a well-established early DDR event that helps recruit repair factors—such as BRCA1, the MRN complex, and 53BP1—to sites of DNA damage (25, 26). This mark is largely installed by ATM and ATR, which also expand the DDR program by phosphorylating additional repair and checkpoint components (27, 28). Notably, H2AX can also be modified on serine/threonine residues by O-GlcNAc, and multiple studies point to a reciprocal relationship between these modifications: higher phosphorylation can dampen O-GlcNAcylation, and vice versa (29–31). In several experimental systems, γH2AX-associated O-GlcNAcylation appears to act as a local “brake,” limiting how far DDR signaling spreads from damaged chromatin into neighboring undamaged regions. This fits better with an OGT-dependent tuning of signal amplitude than with a uniformly pro-repair role in every context (21, 23, 32).

**Figure 1:**
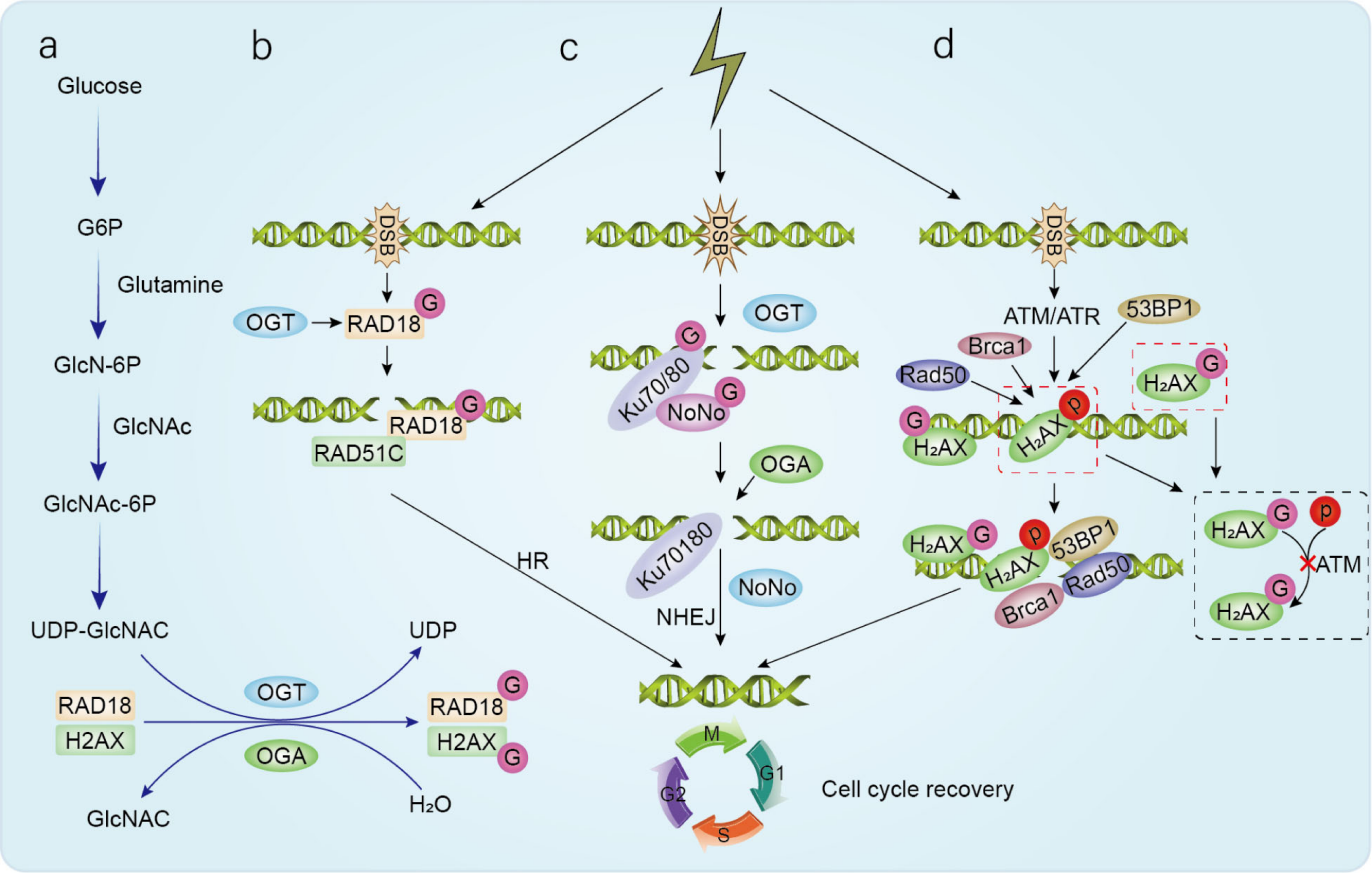
The role of glycosylation in radiotherapy-induced DNA damage. **(a)** The hexosamine biosynthesis pathway provides the sugar substrate for O-GlcNAcylation. OGT catalyzes the addition of sugar chains to nuclear and cytoplasmic proteins, while O-GlcNAcase catalyzes the removal of the sugar. **(b)** O-glycosylated RAD18 promotes RAD51 binding to damaged DNA during HR, facilitating its recruitment and repair at DNA damage sites. **(c)** OGA recognizes O-GlcNAcylated substrates, including NONO and Ku complexes. OGA deglycosylates substrates like NONO, aiding timely NONO removal for DSB repair. **(d)** Following DNA damage, ATM/ATR phosphorylates H2AX. Phosphorylated γH2AX recruits DNA repair factors, such as Rad50, 53BP1, and BRCA1, to the DNA damage site, thereby coordinating DNA repair mechanisms and cell cycle checkpoints to maintain genomic integrity. Conversely, O-GlcNAcylation of H2AX blocks ATM-dependent H2AX phosphorylation, thereby restricting the spread of phosphorylation from DNA damage sites to undamaged regions.

Within TLS, OGT-dependent O-GlcNAcylation of the E3 ubiquitin ligase RAD18 has been linked to CDC7-driven phosphorylation of RAD18 (Ser434), to damage-induced PCNA monoubiquitination, and to Polη focus formation—steps that are central to lesion bypass (33, 34). Consistent with this, blocking RAD18 O-GlcNAcylation reduces PCNA monoubiquitination, compromises TLS, and increases radiosensitivity (33, 34). RAD18 O-GlcNAcylation has also been reported to support RAD51 loading/engagement at damaged DNA, suggesting one route by which TLS-related processing interfaces with HR pathway choice or coordination (35) (**Figure 1**).

A related HR example involves the m6A reader YTHDC1: following DNA damage, YTHDC1 becomes O-GlcNAcylated and, in the reported models, promotes HR-dependent repair and survival by enhancing RAD51 recruitment to breaks (36). Taken together, these studies support the view that O-GlcNAc cycling can help coordinate TLS–HR decisions and fine-tune DDR signaling on chromatin.

#### 2.1.2 NHEJ: OGA-driven de-O-GlcNAcylation and NONO/Ku dynamics on chromatin

In the context of NHEJ, the key theme that emerges is OGA-dependent removal of O-GlcNAc as a regulator of repair-factor behavior on chromatin. Reports suggest that the pseudo-HAT domain of OGA can engage NHEJ-associated components, including NONO and the Ku70/80 complex, with consequences for NHEJ initiation and/or progression (37, 38). Consistent with this model, pharmacologic or genetic suppression of OGA extends the residence time of NONO at DNA damage sites and slows its turnover on chromatin, which in turn compromises NHEJ and enhances radiation-induced cell killing in experimental systems (39).

#### 2.1.3 Checkpoint control and cell-cycle gating: O-GlcNAc at CDK/CKI nodes

Because HR, NHEJ, and TLS are gated by cell-cycle phase, checkpoint and cell-cycle regulators provide another route through which O-GlcNAc can shape DDR outcomes. One example is the CDK inhibitor p27: its O-GlcNAcylation has been associated with changes in phosphorylation, increased cytoplasmic accumulation, and accelerated turnover, with downstream effects on cyclin/CDK activity and cell-cycle progression that, in some settings, are linked to radioresistance (40–42). O-GlcNAcylation has also been reported to stabilize cyclin D1 by slowing its ubiquitination (43). Taken together, these observations suggest that O-GlcNAc can influence DDR not only at DNA breaks, but also upstream through cell-cycle control that helps determine HR versus NHEJ usage.

#### 2.1.4 Limitations of current evidence and priorities for validation

Much of the mechanistic work connecting O-GlcNAc to DDR comes from *in-vitro* irradiation studies in established cancer cell lines, typically using single acute doses and short sampling windows (minutes to hours). Interpretation can also be complicated by heavy reliance on genetic perturbations or tool inhibitors that are not always fully specific. Another point is directionality: O-GlcNAc is not uniformly “pro-repair” or “anti-repair.” Depending on timing, chromatin context, and the endpoint measured, it can support repair by stabilizing repair assemblies, or it can dampen DDR signaling—such as by limiting γH2AX spread. Future progress will benefit from designs that better mirror clinical radiotherapy, including fractionated regimens, broader sampling across tumor lineages, and validation in patient-derived systems. Finally, dose- and time-resolved (glyco)proteomic profiling before and after RT would help pinpoint when and where O-GlcNAc changes occur, and how those dynamics track with repair capacity.

### 2.2 The emerging role of N-glycosylation in DNA damage response

Although studies on glycosylation mechanisms affecting DNA damage and repair have focused mainly on O-glycosylation, N-glycosylation also plays a vital role in regulating DNA damage and repair processes. It can ensure the effectiveness and precision of the repair process by promoting the expression of DNA damage repair-related genes, regulating protein stability, and subcellular localization, among other mechanisms.

Because N-glycosylation is largely restricted to the ER–Golgi secretory pathway and is most prominent on secreted and cell-surface proteins, its influence on the DNA damage response is often indirect. Changes in N-glycan architecture can nonetheless reshape DDR-relevant inputs by tuning receptor-driven signaling, protein trafficking, and microenvironmental interactions, which ultimately converge on canonical DDR nodes such as ATM, ATR, and DNA-PK and on checkpoint control. Consistent with this idea, radiation-associated shifts in terminal sialylation—including ST6GAL1-linked programs—have been associated with therapy resistance and may help sustain repair and survival by modulating integrin and receptor tyrosine kinase signaling (40). Together, these observations point to an underappreciated route through which extracellular glycan remodeling can influence intracellular DDR competence.

Patient-informed studies also suggest that upregulation of sialyltransferases may support adaptation to oxidative stress and ER stress after radiotherapy. In triple-negative breast cancer, higher ST3GAL4 expression correlates with radioresistance and poorer outcomes, and mechanistic work indicates that ST3GAL4-dependent sialylation of the ER chaperone HSP90B1 can bolster proteostasis by improving clearance of misfolded proteins and limiting ROS accumulation (41). Although this does not represent a classic “DDR protein” modification, it illustrates how N-glycan remodeling can shift cellular stress states in ways that ultimately affect DDR efficiency and radiosurvival.

A related case involves CDON, a cell-adhesion-associated oncogenic regulatory protein that is glycosylated and has been implicated in DNA damage repair in cardiomyocytes (42). N-glycosylation of CDON has been proposed to support repair by maintaining expression of DDR-associated genes and limiting damage accumulation, whereas mutation of CDON glycosylation sites weakens these protective effects (43). Whether a comparable CDON–DDR axis operates in irradiated tumor models remains an open question that warrants systematic testing.

N-glycosylation of the immune checkpoint PD-L1 provides another possible link between glycan remodeling, immune escape, and cell-intrinsic radioresistance. PD-L1 suppresses T-cell-mediated antitumor immunity through engagement of PD-1 (44) and is a major target of immunotherapy (45). PD-L1 is extensively N-glycosylated, and this modification can stabilize the protein, favor its retention at the plasma membrane, and enhance PD-1 binding, thereby strengthening immune evasion (46). Importantly, how radiation intersects with PD-L1 glycosylation appears to vary across contexts. Shu and colleagues reported that ionizing radiation induces site-specific PD-L1 deglycosylation, which promotes nuclear translocation and recruitment to double-strand break sites; in that setting, nuclear PD-L1 interacted with Ku70/80 and supported NHEJ and radio-tolerance (47, 48). PD-L1 has also been proposed to function as an RNA-binding protein that stabilizes DDR-related transcripts such as NBS1 and BRCA1 (49). By contrast, work in TNBC showed that D-mannose can trigger AMPK-dependent phosphorylation of PD-L1 at S195, leading to aberrant glycosylation, proteasomal degradation, and reduced stability of DDR-associated mRNAs, ultimately sensitizing tumors to radiotherapy (48).

Reconciling these seemingly divergent conclusions requires close attention to experimental context. The reported effects come from different tumor types (for example, lung cancer versus TNBC), employ different irradiation regimens and sampling windows, and emphasize distinct readouts—ranging from site-specific glycosylation and trafficking to nuclear functions. Moreover, radiotherapy can drive both rapid DDR rewiring and slower, senescence-associated programs, making it unlikely that PD-L1 glycosylation remains constant over time. One plausible model is that early changes relate to trafficking and repair, whereas later phases reflect senescence-linked increases. These considerations underscore the need for dose- and time-resolved analyses in well-matched systems.

## 3 Glycosylation in radiotherapy-induced immune responses: implications for immunotherapy

Beyond the DNA damage response, glycosylation also influences how tumors respond to irradiation by shaping immune-checkpoint function and innate-immune signaling in the tumor microenvironment (50, 51). Radiotherapy can boost antitumor immunity—for example by triggering immunogenic cell death and increasing antigen availability—but it can also drive compensatory immune escape, such as induction of PD-L1 (52–54). Since glycan remodeling affects both checkpoint stability/engagement and innate pathways including cGAS–STING, it is important to interpret glyco-immune findings in the context of radiotherapy conditions (dose, fractionation, and tumor type) when evaluating mechanisms and designing effective radio-immunotherapy combinations (55).

### 3.1 PD-L1 glycosylation in tumor immune evasion and radiotherapy resistance

Clinical studies have shown that radiotherapy can induce upregulation of PD-L1 expression through multiple pathways, and thus, combining radiotherapy with anti-PD-1/PD-L1 antibodies has been shown to improve the clinical prognosis of many cancers (56). From a glycosylation perspective, radiotherapy-induced PD-L1 upregulation may be associated with altered glycosylation modifications that affect the expression level and stability of PD-L1 and regulate its functional status (57). In addition, modified glycosylation modifications can also affect the affinity of PD-L1 for PD-1, which further affects the immune escape ability of tumor cells and the response of patients to immunotherapy (58–60). Thus, radiotherapy-induced upregulation of PD-L1 can enhance the immunosuppressive function of PD-L1 by altering its glycosylation status.

#### 3.1.1 Effect of glycosylation on the stability of PD-L1

PD-L1 carries abundant N-linked glycans, which support its folding and stability and help maintain its presentation at the cell surface, thereby strengthening checkpoint activity (61–63). Radiotherapy can alter PD-L1 glycosylation, but the direction and mechanism vary with context. In therapy-induced senescence models, ionizing radiation increases PD-L1 transcription and promotes N-glycosylation by upregulating ribophorin 1 (RPN1) and other OST-B components, leading to greater PD-L1 stability and membrane localization (64, 65). By contrast, in some tumor models acute irradiation has been linked to site-specific PD-L1 deglycosylation and nuclear trafficking, a pattern associated with proposed roles in double-strand break repair (47, 48). Metabolic stressors such as 2-deoxyglucose (2-DG) can also trigger PD-L1 deglycosylation and reshape immune suppression in TNBC settings (66). Taken together, these data suggest that PD-L1 glyco-regulation depends strongly on radiotherapy conditions, including dose and fractionation, as well as tumor type and the time point at which samples are collected.

#### 3.1.2 Glycosyltransferases in the regulation of PD-L1 Stability

**Figure 2:**
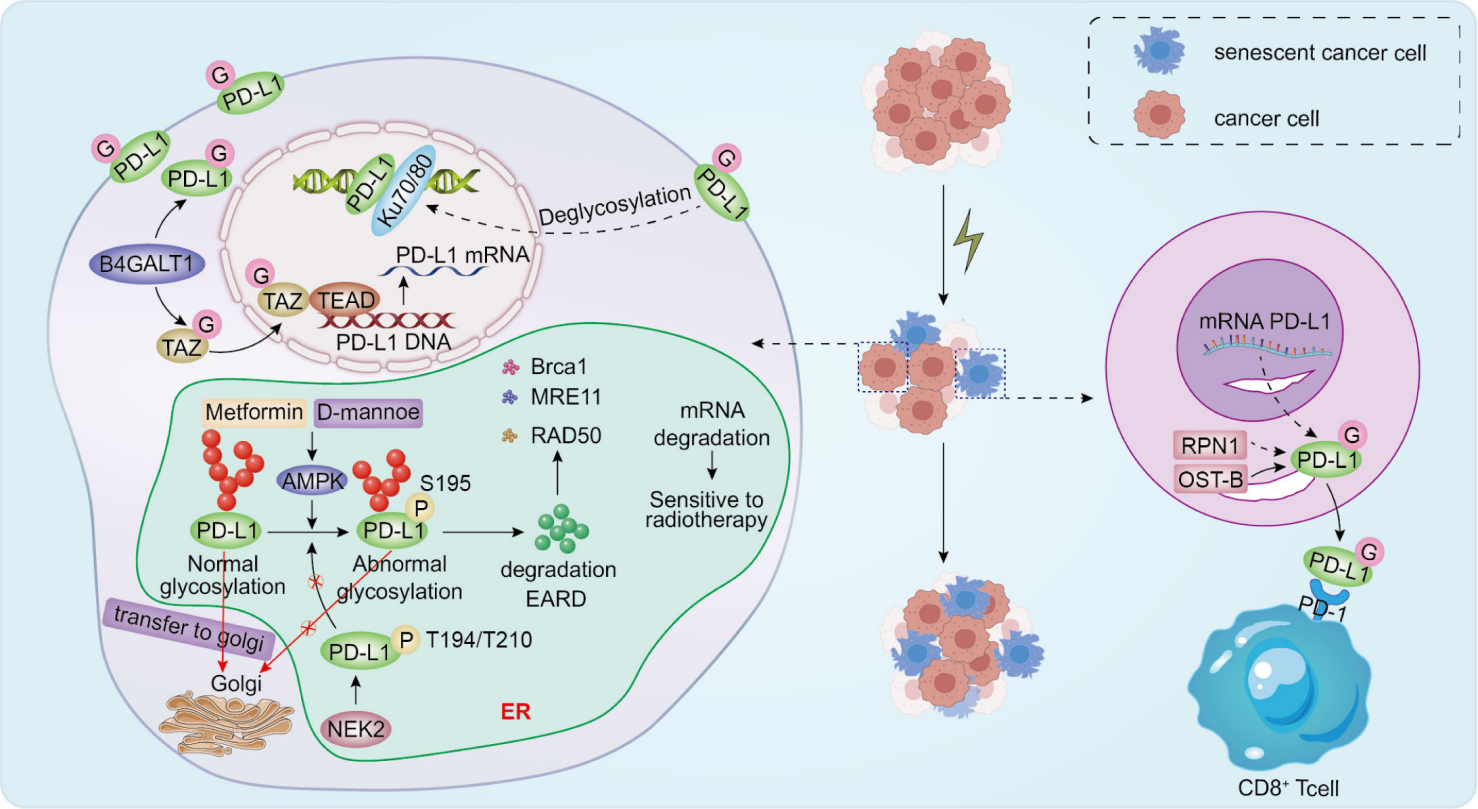
IR can induce cancer cell senescence by increasing PD-L1 transcription while also promoting PD-L1 glycosylation through elevated expression of RPN1 and other OST-B complex components, thereby stabilizing PD-L1 and facilitating its translocation to the cell membrane. At the same time, IR can cause PD-L1 deglycosylation, leading to its translocation from the membrane to the nucleus and recruitment to DNA double-strand break (DSB) sites. PD-L1 recruited to DSB sites accelerates NHEJ-mediated DNA double-strand break repair, leading to radiation resistance. D-mannose and metformin activate AMPK, causing PD-L1 phosphorylation at position S195. This leads to abnormal PD-L1 glycosylation and proteasomal degradation, as well as mRNA instability in DNA damage repair-related genes such as BRCA1, RAD50, and MRE11, thereby sensitizing tumor cells to IR treatment.

Glycosyltransferases play a key role in maintaining the protein stability and immunosuppressive function of PD-L1 by catalyzing its N-linked glycosylation modification (67). The recently discovered role of the glycosyltransferase B4GALT1 in lung adenocarcinoma (LUAD) is of great interest.B4GALT1 not only stabilizes the PD-L1 protein by regulating N-glycosylation in LUAD, but also has a significant effect on the mRNA level of PD-L1. It stabilizes TAZ protein through glycosylation, and TAZ protein interacts with the transcription factor TEAD, which in turn promotes PD-L1 transcription (68). Therefore, the combined application of glycosylation inhibitors and radiotherapy may provide a new effective strategy for tumor treatment. RNA-binding protein RBMS1, on the other hand, regulates the mRNA stability of B4GALT1, which is elevated in breast cancer, and its absence disrupts the mRNA stability of B4GALT1, inhibits PD-L1 glycosylation, and promotes ubiquitylated degradation of PD-L1 (69)(**Figure 2**). Moreover, RBMS1 depletion leads to inhibition of translation of the iron death repressor SLC7A11, and reduced SLC7A11 promotes iron death, thereby inhibiting lung cancer progression and sensitizing drug-resistant cancer cells to radiotherapy (70). Therefore, targeted inhibition of RBMS1 is expected to be a new tumor treatment strategy with potential clinical applications.

#### 3.1.3 EGFR and PD-L1 glycosylation

In glioblastoma (GBM), chromosome 7p11 amplification leads to increased EGFR copy number in GBM tumors, the most common alteration in primary GBM (59). However, targeting EGFR is usually inefficient, and the underlying study mechanisms remain unclear. Recent studies have found that SEC61G is always co-amplified with EGFR and highly expressed in GBM. SEC61G promotes translocation and glycosylation of PD-L1 to the endoplasmic reticulum, and its depletion promotes enhanced CD8+ T activity, which inhibits the development of GBM. Therefore, SEC61G may lead to reduced sensitivity to radiotherapy by promoting tumor immune escape mechanisms, suggesting that targeting SEC61G is expected to be a new strategy for the combination therapy of EGFR-amplified GBM (71).

#### 3.1.4 Effect of phosphorylation modification of PD-L1 on glycosylation

Phosphorylation modifications similarly regulate the stability of PD-L1, as phosphorylation induces aberrant glycosylation of PD-L1, thereby altering its stability (72). Metformin has been shown to play an important role in antitumor and immune responses. As the main effector of metformin (73), when metformin activates AMPK in the ER lumen, AMPK can directly phosphorylate the S195 site of PD-L1. Phosphorylation of the S195 site induces aberrant PD-L1 glycosylation and blocks its translocation from the ER to the Golgi, leading to PD-L1 accumulation in the ER and ER-associated degradation (ERAD), thereby increasing CTL activity against cancer cells (74). Based on the above studies, Zhang et al. suggested that TNBC D-mannose could activate AMPK and phosphorylate PD-L1 at the S195 site, leading to aberrant glycosylation and proteasomal degradation of PD-L1. In addition, D-mannose-induced PD-L1 degradation also leads to mRNA instability of DNA damage repair-related genes, which enhances the sensitivity of breast cancer cells to radiation therapy and is expected to improve the efficacy of radiation therapy in TNBC patients (49) (**Figure 2**).

NEK2 is a typical cell cycle regulatory protein that plays a vital role in cell cycle and mitotic regulation, and its elevated expression promotes tumorigenesis through aberrant cell cycle regulation (75, 76). Studies have shown that NEK2 can mediate the phosphorylation of the PD-L1 T194/T210 site, and its phosphorylation may interrupt S195-induced aberrant PD-L1 glycosylation and prevent its degradation in the endoplasmic reticulum lumen via the ubiquitin proteasome pathway, thereby promoting normal PD-L1 glycosylation to maintain its stability (77). In addition, knockdown of NEK2 downregulated the mRNA and protein levels of Wnt1 and inhibited the activation of the Wnt/β-catenin signaling pathway, suppressing tumorigenesis and enhancing radioresistance in cervical cancer (78). Based on the dual role of NEK2 in cell cycle regulation and tumor immune microenvironment remodeling, developing small molecule inhibitors targeting NEK2 has become a current research hotspot for combined radiotherapy and immunotherapy (76).

### 3.2 CD24/Siglec-10 signaling and radiotherapy resistance

Siglecs are immunoglobulin-like lectins that recognize sialic-acid-containing glycans and, through these interactions, can shape antitumor immunity (79). Many tumors display increased or aberrant sialylation, creating abundant sialoglycans that engage Siglec receptors on immune cells and establish an immunosuppressive “sialic acid–Siglec axis.” Activation of this axis dampens immune effector functions and supports immune escape (80). For example, elevated Siglec expression on T cells has been associated with T-cell dysfunction and reduced tumor-killing capacity (81). These observations raise the possibility that Siglec-dependent immune suppression may also contribute to resistance to radiotherapy. Accordingly, approaches that interfere with sialoglycan biosynthesis or block sialoglycan–Siglec binding could help restore immune activity and improve radiosensitivity (81, 82). Siglecs have therefore become attractive candidates for radioimmunotherapy strategies that aim to relieve glyco-immune checkpoints.

**Figure 3:**
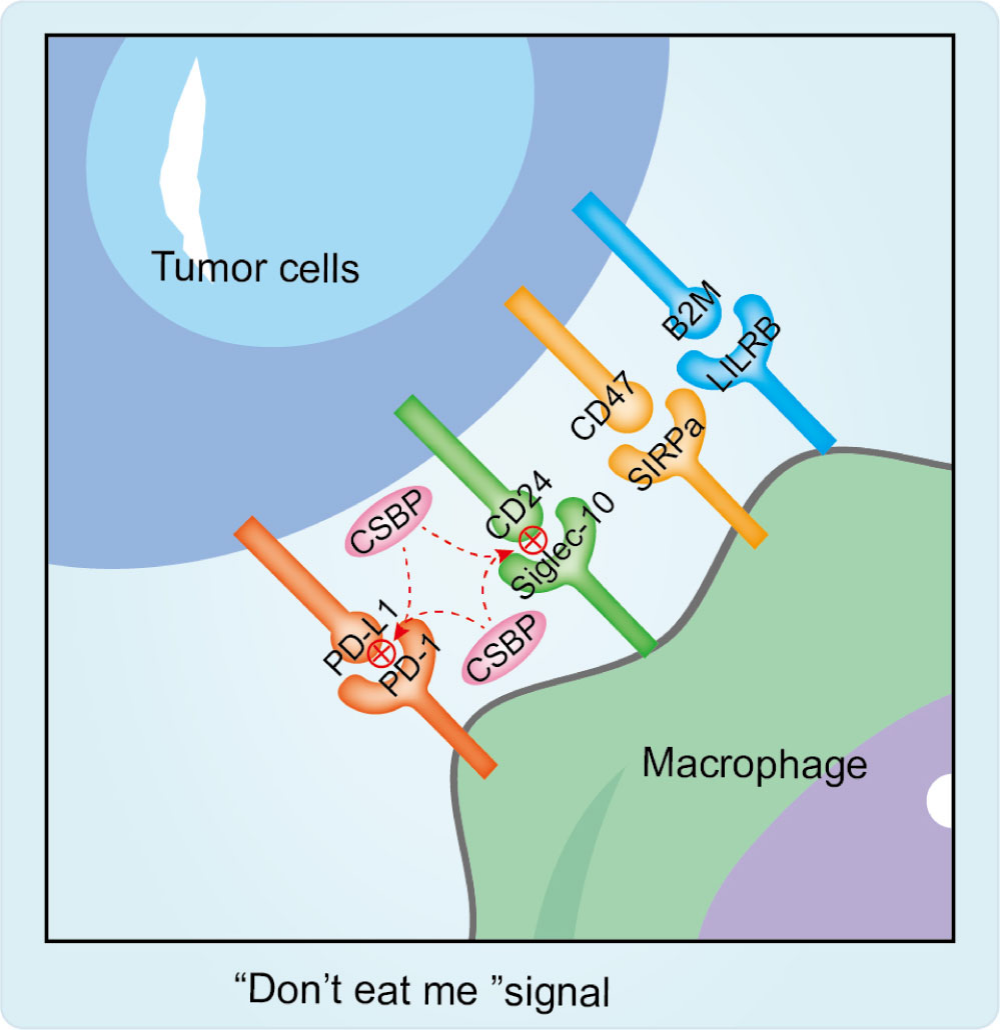
“Don’t Eat Me” signaling. Classic “Don’t Eat Me” Signaling Molecules include the CD74-SIRPa axis, CD24-Siglec-10 axis, B2M-LILRB axis, and PD-L1-PD-1 axis. Notably, the anti-hydrolytic blocking peptide CSBP not only inhibits CD24/Siglec-10 interactions but also blocks PD-1/PD-L1 activity. Furthermore, combining RT with CSBP enhances antitumor efficacy.

Siglec-10 is a broadly expressed inhibitory receptor found on several immune populations, including subsets of tumor-associated macrophages (TAMs), dendritic cells, NK cells, and activated T cells (83, 84). In sialylation-high tumors, sialoglycans can engage Siglec-10 (and other Siglecs) to reinforce the immunosuppressive sialic acid–Siglec axis (79–81). Among the best-characterized examples is the CD24–Siglec-10 pathway, which delivers a “don’t-eat-me” signal to macrophages and, in certain tumor contexts, can also impair NK-cell activity (85, 86). A CD24/Siglec-10 blocking peptide (CSBP) has shown preclinical synergy with radiotherapy, consistent with the idea that RT-driven antigen release may translate into stronger immunity when glyco-immune inhibitory signals are lifted (87) (**Figure 3**).

Siglec-15 has also emerged as a glyco-immune checkpoint of interest. It binds sialylated ligands and can suppress antitumor T-cell responses. A humanized anti–Siglec-15 antibody (NC318) has entered early clinical evaluation (phase I/II; for example NCT03665285 and NCT04699123) (88). Although combinations with radiotherapy have not yet been examined systematically, these studies highlight the clinical feasibility of targeting glycan checkpoints and point to an important unanswered question: whether radiotherapy alters Siglec-15 expression or reshapes the availability of Siglec-15 ligands within the tumor microenvironment.

### 3.3 Signaling mechanisms of glycosylation in immune escape

DNA damage can lead to the accumulation of cytosolic DNA, which in turn triggers cGAS–STING signaling and type I interferon production, helping to boost antitumor immunity after radiotherapy (89, 90). Glycosylation can modulate this pathway. In several tumor settings, increased OGT activity and elevated O-GlcNAcylation have been linked to dampened innate immune activation, whereas pharmacologic or genetic inhibition of OGT has been reported to increase DNA damage and cytosolic DNA, activate cGAS–STING, and promote dendritic-cell activation and CD8+ T-cell responses (91–93). Together, these findings provide a rationale for combining OGT inhibition with RT—and potentially with PD-1/PD-L1 blockade—particularly in contexts where innate immune priming is a major determinant of therapeutic response (55).

Much of the mechanistic work linking glycosylation to radioimmunology still comes from simplified experimental systems, such as *in vitro* irradiation of tumor cells and syngeneic mouse models. By contrast, patient-level evidence remains relatively sparse and is often limited to correlative readouts—such as glycosyltransferase expression signatures or PD-L1 immunohistochemistry—rather than direct, site-specific measurements of glycosylation. For meaningful clinical translation, it will be important to use more comparable radiotherapy conditions across studies and to incorporate patient-derived organoids, immunocompetent models, and matched pre- and post-RT specimens whenever possible.

## 4 The impact of glycosylation on the tumor microenvironment during radiotherapy

The tumor stroma is a heterogeneous ecosystem of non-malignant cells and extracellular-matrix (ECM) components, including fibroblasts, immune cells, vascular endothelial cells, adipocytes, and structural matrix proteins (1, 94). Radiotherapy perturbs this compartment by inducing vascular injury, inflammatory signaling, hypoxia, and pro-fibrotic remodeling. Many of these stromal responses are influenced by glycosylation of adhesion molecules, growth-factor receptors, and ECM proteins. That said, direct, subset-specific evidence that radiation rewires glycosylation programs in stromal cells is still limited and often depends on the model system used. This makes it important to distinguish findings from *in vitro* endothelial assays from those obtained *in vivo* and, especially, in patient-based studies.

### 4.1 Radiotherapy-induced changes in endothelial cell adhesion and their relationship to glycosylation modifications

Tumor angiogenesis is a major determinant of oxygen delivery and, consequently, radiotherapy response, with endothelial cells serving both as direct radiation targets and as key regulators of immune-cell entry into tumors (95, 96). Leukocyte recruitment depends on sequential steps—rolling, firm adhesion, and transmigration—that are tightly controlled by glycosylated adhesion systems, including selectins with their sialylated ligands and molecules such as ICAM-1 and VCAM-1 (97, 98). Ionizing radiation can drive endothelial dysfunction and senescence, often reflected by increased permeability, loss of attachment to the basement membrane, and apoptosis. These changes can impair perfusion and, in some settings, contribute to tumor control (99, 100). At the same time, endothelial activation and senescence after irradiation can reprogram local inflammatory signaling (101). While endothelial inflammatory phenotypes after IR have been described extensively, far fewer studies have directly examined how radiotherapy alters the glycosylation of endothelial adhesion molecules and their ligands—features such as N-glycan branching or terminal sialylation and fucosylation that can directly tune leukocyte–endothelium interactions. Addressing this gap will likely require pairing glycoproteomic profiling with functional trafficking assays under well-defined, clinically relevant RT regimens to determine whether endothelial glycan remodeling favors antitumor immunity or instead promotes chronic inflammation and fibrosis.

All endothelial cell adhesion molecules involved in leukocyte migration are heavily N-glycosylated, yet the potential of N-glycosylation to control the adhesion function of endothelial adhesion molecules has rarely been investigated (102). This may be due to the fact that endothelial cell adhesion molecules, which regulate leukocyte migration, are typically not expressed in quiescent cells. Furthermore, N-glycan processing is significantly inhibited during inflammation and cellular dysfunction (103). Recent studies have shown that ionizing radiation alters the vascular network and increases the expression of endothelial cell adhesion molecules, specifically high-mannose-type N-glycans (104). These high-mannose N-glycans and other glycans may act as ligands for monocyte adhesion and regulate endothelial signaling (105). This suggests that radiation can affect immune cell recruitment in healthy tissues and tumors by altering the form of endothelial cell glycosylation. Moreover, ionizing radiation causes alterations in N- and O-glycosylation genes to affect endothelial cell function (105). Overall, endothelial cell glycosylation may be a key target in chronic inflammatory responses or involved in tumor control by radiation therapy. Targeted therapy against the glycosylation of endothelial cell adhesion molecules is important for future tumor control.

### 4.2 Radiotherapy-induced remodeling of the extracellular matrix and regulation of glycosylation

The extracellular matrix (ECM) undergoes profound remodeling in tumors, and RT-associated inflammation and fibrotic programs can further reshape this compartment. Relative to normal tissue, tumor ECM typically shows greater deposition, increased cross-linking, and altered glycosylation—features that can stiffen the matrix and reprogram mechanotransduction (106). ECM stiffening can be driven by both non-enzymatic glycation, through formation of advanced glycation end products (AGEs), and enzymatic glycosylation of ECM proteins and proteoglycans. For instance, exposing type I collagen gels to reducing sugars such as glucose-6-phosphate promotes AGE formation and increases matrix cross-linking (106, 107). A stiffer matrix can strengthen pro-survival integrin signaling and may also impede oxygen and drug delivery, both of which could reduce RT effectiveness. Clinically, liver tumors arising in cirrhotic (fibrotic) livers have been reported to respond less well to radiotherapy (108, 109), supporting the concept that a fibrotic ECM can shape RT response. However, studies that track ECM glycation/glycosylation over time after RT and directly test its causal contribution to radioresistance remain limited.

## 5 Metabolic and glycosylation modifications synergistically regulate radiotherapy resistance

Radiotherapy can provoke an immediate metabolic stress response—manifesting as mitochondrial impairment, redox disruption, and reduced nutrient availability—and it can also lead to longer-term metabolic rewiring. Because nucleotide-sugar biosynthesis depends on substrate supply, these metabolic changes can be transmitted directly to glycosylation programs. The hexosamine biosynthetic pathway (HBP) is a central link in this connection: by channeling glucose and glutamine into UDP-GlcNAc production, it couples metabolic flux to both O-GlcNAcylation and N-glycan branching. Importantly, metabolic adaptations to RT vary with oxygenation, tumor lineage, and fractionation strategy. Studies examining the metabolism–glycosylation–RT interface should therefore report these variables explicitly and, whenever possible, validate key conclusions in patient-derived models.

### 5.1 Metabolic reprogramming and glycosylation

Cancer cells reshape their metabolism to support proliferation and withstand stress, and radiotherapy imposes additional metabolic demands by generating ROS, increasing the energetic cost of DNA repair, and altering the microenvironment—for example through hypoxia and vascular injury (110, 111). RT can acutely impair mitochondrial function and redox balance, and with repeated or sustained exposure it may favor metabolic configurations that better support survival. Because glycosylation depends on nucleotide-sugar supply and is influenced by redox and ER homeostasis, these RT-driven metabolic changes can be translated into shifts in glycosylation. A central conduit is the hexosamine biosynthetic pathway (HBP): changes in HBP flux and UDP-GlcNAc availability directly affect both O-GlcNAcylation and N-glycan branching. Viewed this way, alterations in glycolysis or oxidative phosphorylation are not simply generic cancer traits; under RT they often reflect regimen- and context-specific constraints that reshape metabolite pools and, in turn, enable glycosylation programs associated with radioresistance (112).

At present, direct mechanistic evidence linking radiation-induced metabolic remodeling to glycosylation-dependent regulation remains limited. Many studies infer glycosylation changes from metabolic phenotypes rather than measuring glycan structures directly. Going forward, it will be important to quantify UDP-GlcNAc pools, HBP activity (for example GFAT flux), and site-specific O-GlcNAc and N-glycan changes in matched samples collected before and after irradiation, ideally using clinically relevant fractionated regimens. Such designs would help separate early stress responses (hours) from longer-term adaptations (days to weeks), which may differ in both mechanism and therapeutic opportunity.

Because nucleotide-sugar biosynthesis depends on substrate availability, RT-induced changes in glucose and glutamine utilization can reshape glycosylation by altering flux through the hexosamine biosynthetic pathway (HBP) and the availability of UDP-GlcNAc (113–116). Although only a small fraction of glucose is diverted into the HBP, increased glutamine uptake can further promote UDP-GlcNAc production, thereby supporting both O-GlcNAcylation and N-glycan branching (114, 115). Studies across tumor types, including colon cancer, have linked elevated GFAT/HBP activity to malignant phenotypes and suggest that restricting HBP flux can suppress tumor growth and metastasis (117–119). In the context of radiotherapy, an important limitation is that many reports infer glycan remodeling from metabolic phenotypes; future studies should directly quantify UDP-GlcNAc pools and measure site-specific glycosylation under dose- and time-resolved RT regimens.

Mitochondrial metabolism strongly influences RT response by controlling ATP supply, redox buffering, and irradiation−induced ROS (120). O−GlcNAcylation can rapidly modify mitochondrial proteins, and a catalytically active mitochondrial OGT isoform (mOGT) has been described with expression responsive to nutrient state (121, 122). mOGT loss impairs mitochondrial integrity, whereas mOGT upregulation has been linked to altered respiratory chain activity and oxidative−stress control, potentially via reduced VDAC3 and amplified ROS production (123–125). Whether this ROS shift enhances RT killing or promotes adaptive survival appears model− and timing−dependent, underscoring the need for dose− and time−resolved measurements of mitochondrial function and O−GlcNAc in RT settings (126).

**Figure 4:**
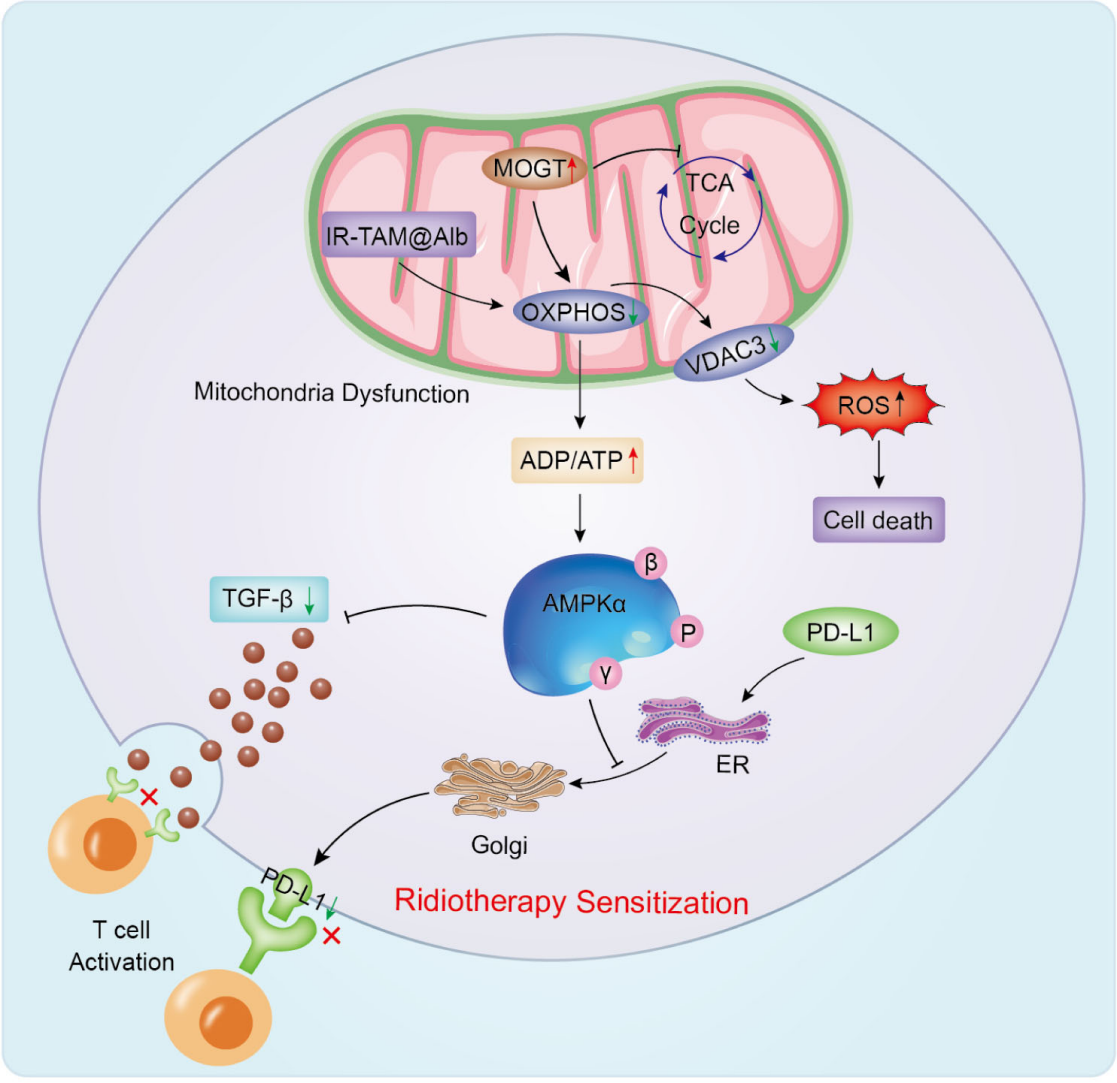
The regulation of mitochondrial metabolism by glycosylation and its role in radiotherapy response. Upregulation of mOGT significantly reduces mitochondrial VDAC3 protein expression by inhibiting the function of respiratory chain complexes. This decrease in VDAC3 expression further exacerbates excessive ROS accumulation, ultimately leading to cell death. Moreover, mOGT upregulation inhibits the rates of OXPHOS and glycolysis, reducing ATP synthesis and ultimately triggering AMPK protein phosphorylation. However, activated AMPK specifically binds PD-L1 and induces phosphorylation at its S195 site, thereby initiating an endoplasmic reticulum-mediated protein degradation pathway that inhibits PD-L1 transport from the endoplasmic reticulum to the Golgi apparatus. Moreover, TGF-β can also be inhibited by AMPK phosphorylation, leading to immune activation. This dual regulatory effect significantly enhances the antitumor immune response. The mitochondrial inhibitor TAM combined with the mitochondrial-targeted drug 7-methylthymidine can synergistically inhibit both PD-L1 and TGF-β signaling pathways.

Mitochondrial state may also influence the immune landscape during radiotherapy by regulating PD-L1 and TGF-β signaling (127). Activation of AMPK has been reported to promote ER-associated degradation of PD-L1 through phosphorylation at S195 and to dampen TGF-β signaling, changes that can collectively tilt the balance toward antitumor immunity (128, 129). In line with this idea, a clinical study combined the mitochondrial inhibitor TAM with a mitochondria-targeting heptamethylcyanine dye, aiming to suppress PD-L1/TGF-β pathways, overcome RT resistance, and lessen treatment-associated fibrosis (130) (**Figure 4**).

Taken together, mOGT is well positioned to influence radiotherapy responses by regulating mitochondrial function, oxidative-stress handling, and downstream metabolic signaling. The available evidence points to strong context dependence: in some models mOGT activity appears to increase radiosensitivity, whereas in others it may facilitate adaptation and resistance. Determining when mOGT represents a therapeutically actionable vulnerability will require further work, ideally in immunocompetent settings and in patient-derived models.

### 5.2 Glycosylation of metabolic enzymes in radiotherapy response

Aerobic glycolysis is frequently associated with tumor radioresistance, and a range of preclinical studies have shown that inhibiting key glycolytic enzymes can enhance radiosensitivity (131). These observations have helped motivate combination strategies that integrate metabolic interventions with radiotherapy (132). One mechanistic lens for understanding this link is O-GlcNAcylation, a nutrient-responsive modification: OGT uses UDP-GlcNAc generated through the hexosamine biosynthetic pathway to modify many signaling and metabolic proteins, thereby translating nutrient availability into stress-adaptation programs. Pyruvate kinase M2 (PKM2) provides a representative example. O-GlcNAcylation of PKM2 can influence its oligomeric state and adjust glycolytic flux, potentially reinforcing—or redirecting—Warburg-like metabolism in ways that affect RT response (133, 134). Taken together, these data highlight the importance of measuring metabolic flux alongside glycosylation under well-defined RT conditions, particularly because oxygen and nutrient availability can differ markedly between *in vitro* systems and *in vivo* tumors.

**Figure 5:**
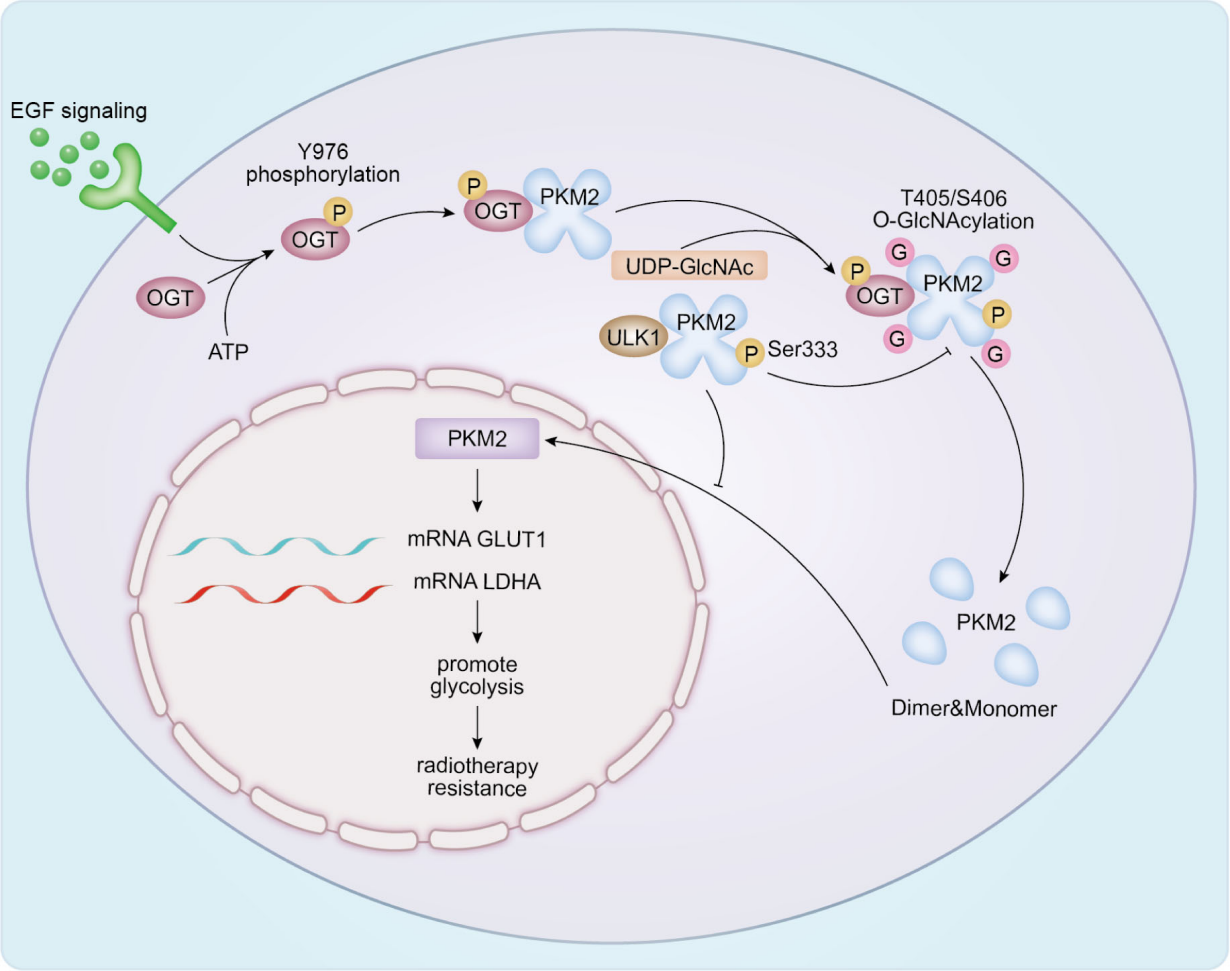
Glycosylation of metabolic enzymes in radiotherapy response. EGF promotes OGT binding to PKM2 by stimulating OGT Y976 phosphorylation, leading to PKM2 T405/S406 site O-GlcNAcylation and tetradisassembly. PKM2 O-glycosylation reduces PKM2 activity, while unstable PKM2 tetramers cause PKM2 to relocalize to the cell nucleus, promoting glucose metabolism. O-GlcNAcylation and depolymerization. PKM2 O-glycosylation reduces PKM2 activity, while unstable PKM2 tetramers cause PKM2 to relocalize to the nucleus, promoting GLUT and LDHA expression, ultimately enhancing glycolysis and leading to radiation resistance. However, ULK1 interacts with PKM2 and phosphorylates its Ser333 site. PKM2-S333 phosphorylation antagonizes PKM2 O-GlcNAcylation, promotes tetramer formation and enzymatic activity, reduces nuclear localization, and suppresses c-Myc expression. By downregulating glucose and lactate production, PKM2 attenuates the Warburg effect, thereby sensitizing cells to radiation therapy.

Pyruvate kinase M2 (PKM2) serves as a key metabolic switch in Warburg-like glycolysis (134). O-GlcNAcylation of PKM2 at T405/S406 has been reported to destabilize the tetramer, lower catalytic activity, and promote nuclear translocation. In the nucleus, PKM2 can induce expression of glycolysis-related genes such as GLUT1 and LDHA, thereby strengthening glycolytic output and contributing to radioresistance. In contrast, ULK1-dependent phosphorylation of PKM2 at S333 appears to counteract O-GlcNAcylation, favor tetramer assembly, and dampen nuclear PKM2/c-Myc signaling. This shift reduces glucose consumption and lactate production and has been associated with increased radiosensitivity (135) (**Figure 5**).

Furthermore, in lung cell carcinoma, phosphofructokinase 1 (PFK1) is the primary regulatory enzyme controlling the flux of the glycolytic pathway (136). Tian et al. found that PFK1 was overexpressed in rectal cancer, and its high expression was negatively correlated with the radiosensitivity of rectal cancer. Inhibition of PFK1 may enhance the radiosensitivity of rectal cancer cells *in vivo* and *in vitro* by inhibiting glycolysis (137). It has been demonstrated that under hypoxic conditions, the Ser529 site of PFK1 undergoes O-GlcNAc glycosylation, which inhibits PFK1 activity. Consequently, this inhibition disrupts the glycolytic process, leading to the accumulation of glycolytic intermediates. This process redirects metabolism to the pentose phosphate pathway, which plays a crucial role in meeting anabolic cellular demands and providing antioxidant defenses. Consequently, increased pentose phosphate pathway flux promotes rapid cell proliferation (138). Therefore, blocking the glycosylation of PFK1 Ser529 not only inhibits tumor formation and cancer cell proliferation but also enhances radiotherapy sensitivity, which may be important in clinical studies.

## 6 Targeted glycosylation drug sensitizing radiotherapy

Preclinical studies support the idea that pharmacologically targeting glycosylation can increase tumor sensitivity to radiotherapy by disrupting DNA-damage response (DDR) signaling, proteostasis and endoplasmic-reticulum (ER) stress adaptation, and the stabilization of immune-checkpoint proteins. Examples include blocking N-glycan processing (e.g., the α-mannosidase inhibitor swainsonine) (139), disrupting the initiation or extension of mucin-type O-glycosylation (e.g., benzyl-α-GalNAc) (140), and inhibiting O-GlcNAc cycling (for instance, OSMI compounds targeting OGT), which in model systems can weaken repair signaling and amplify radiation-induced cell death (141).

However, translating glycosylation-targeting radiosensitizers into the clinic faces practical barriers. Core glycosylation pathways are essential in normal tissues, so broad inhibitors (e.g., tunicamycin) may trigger systemic ER stress and dose-limiting toxicity (142–144). Many available compounds have limited target specificity, and compensatory pathways may blunt efficacy. Drug delivery is also challenging in hypoxic or fibrotic tumors; tumor-targeted carriers, antibody-guided delivery, or intratumoral administration may improve selectivity. Clinical maturity varies: some agents (e.g., swainsonine) have entered early clinical testing, whereas many OGT/OST-directed inhibitors remain preclinical (145) (**Table 2**).

**Table 2:** Summary of glycosylation inhibitors.

Drug name	Target type	Main mechanism of action	Applicable cancer types	Radiotherapy combination	References
Tunicamycin	N-glycosylation inhibitor (OST inhibitor)	Blocks the first step of N-glycan synthesis by inhibiting oligosaccharyltransferase (OST), induces UPR and ER stress, leading to apoptosis	Breast cancer, pancreatic cancer, thyroid cancer	Yes	(146–149)
OSMI-1/OSMI-2	OGT inhibitor	Inhibits O-GlcNAc modification, impairs DNA repair capacity, enhances radiosensitivity	Lung cancer, breast cancer, colorectal cancer, liver cancer	Yes	(150–152)
D-Mannose	AMPK activator	Induces abnormal PD-L1 glycosylation and degradation, enhances radiosensitivity	Triple-negative breast cancer (TNBC)	Yes	(48)
Castanospermine	α-glucosidase inhibitor	Inhibits α-glucosidase, leads to incomplete CD44 glycosylation, preventing proteases (MT1-MMP/ADAM10) from cleaving CD44	Breast cancer	No	(153)
Benzyl-α-GalNAc	O-glycosylation inhibitor	Blocks O-glycan elongation, induces aberrant glycosylation (e.g., Tn antigen accumulation)	Gastric cancer, colorectal cancer, melanoma	No	(154, 155)
Kifunensine	α-mannosidase I inhibitor (ER)	Prevents processing of N-glycans from high-mannose to complex type, resulting in immature glycans	Ovarian cancer	No	(156)
Swainsonine	α-mannosidase II inhibitor	Disrupts complex N-glycan branching, inhibits cancer cell metastasis, and enhances cytotoxic activity of immune effector cells	Esophageal cancer, cervical cancer	No	(157)

## 7 Discussion

A more granular view of how radiotherapy (RT) intersects with glycosylation helps explain why radioresistance emerges, but the evidence base remains early and heterogeneous. Across tumor types, several glycosylation programs—including N-glycosylation, mucin-type O-glycosylation, and O-GlcNAcylation—change following irradiation. Importantly, reported directionality and kinetics vary with dose and fractionation, as well as with the timing and compartment of sampling. Many “conflicting” findings across studies likely reflect differences in models, regimens, and sampling windows rather than true biological incompatibility. Because glycosylation interfaces with multiple pillars of RT response—DNA damage signaling and chromatin regulation, immune regulation (including checkpoints and antigen presentation), stromal and vascular remodeling, and metabolic adaptation—actionable vulnerabilities are more likely to emerge from context-aware, network-level analyses than from single-pathway explanations.

### 7.1 Interpreting glycosylation–RT studies: context, apparent contradictions, and evidence tiers

Across the literature, glycosylation has been linked to both increased and decreased radiosensitivity, and apparent contradictions are frequent. In practice, differences often map to (i) the modification under study (e.g., O-GlcNAcylation versus secretory-pathway N-glycosylation or terminal sialylation), (ii) the compartment being interrogated (nuclear repair assemblies versus cell-surface immune receptors), and (iii) experimental context, including tumor lineage, oxygenation and nutrient availability, and whether irradiation is delivered as a single high dose or via clinically relevant fractionation. PD-L1 offers a useful example: glyco-dependent stabilization of surface PD-L1 can support immune escape in therapy-induced senescence, whereas in some settings acute, site-specific deglycosylation has been associated with nuclear PD-L1 activities that promote NHEJ. Reconciling these observations will require matched, dose- and time-resolved studies and clear separation of immune-mediated endpoints from cell-intrinsic repair readouts.

To sharpen inference, it is helpful to distinguish evidence tiers. Cell culture systems enable mechanistic dissection but can overstate effect sizes and underestimate microenvironmental constraints. Syngeneic and xenograft models incorporate stromal and immune interactions, yet outcomes remain regimen- and context-dependent. Patient datasets are currently dominated by correlative signals (expression-based signatures) and only rarely include paired, site-resolved glycoproteomics before and after RT. Paired patient specimens, patient-derived organoids, and *in vivo* fractionation models will therefore be important for testing causality and for prioritizing clinically tractable biomarkers.

From a translational standpoint, most glycosylation-targeting approaches are still preclinical and face familiar limitations. Core glycosylation machinery is indispensable in normal tissues, raising the likelihood of on-target toxicity. Many inhibitors also lack optimal specificity, and compensatory routes within glycosylation networks remain incompletely defined. In addition, drug penetration is often poor in hypoxic or fibrotic tumors. Progress will likely depend on identifying tumor-selective dependencies (for example, lineage-restricted glycosyltransferases), deploying biomarkers to guide combination selection, and improving delivery to widen the therapeutic window (e.g., tumor-targeted nanocarriers or local administration within RT fields). Ultimately, prospective clinical testing of combination regimens—RT plus glycosylation modulation, with or without immunotherapy or metabolic interventions—will be needed to establish safety and clinical benefit.

Key priorities include integrating glycomics and glycoproteomics with spatial omics and single-cell profiling in matched pre- and post-RT samples; standardizing reporting of radiation regimens and evidence levels (cell culture vs. animal models vs. patient data); and determining whether glycosylation-based biomarkers can reproducibly stratify patients for precision RT combinations.
